# Cognitive Impairment in Prostate Cancer Patients Receiving Androgen Deprivation Therapy: A Scoping Review

**DOI:** 10.3390/cancers17152501

**Published:** 2025-07-29

**Authors:** João Vasco Barreira, Pedro Barreira, Gil Falcão, Daniela Garcez, Pedro Silva, Gustavo Santos, Mário Fontes-Sousa, José Leão Mendes, Filipa Reis, Carla F. Santos, Filipa Ribeiro, Manuel Luís Capelas

**Affiliations:** 1 Universidade Católica Portuguesa, Faculty of Health Sciences and Nursing, Centre for Interdisciplinary Research in Health (CIIS), 1649-023 Lisboa, Portugal; 2 Medical Oncology Department, CUF Oncologia, 1998-018 Lisbon, Portugal; 3 Family Medicine Department, CUF Miraflores, 1495-190 Miraflores, Portugal; 4 Urology Department, Unidade Local de Saứde São José, 1169-050 Lisbon, Portugal; 5 Neuro-Oncology Department, CUF Oncologia, 1099-023 Lisbon, Portugal; 6 Radiotherapy Department, Unidade Local de Saứde Santa Maria, 1649-035 Lisbon, Portugal; 7 Psychiatry Department, Unidade Local de Saúde de Santo António, 4149-003 Porto, Portugal; 8 Medical Oncology Department, Unidade Local de Saứde São Francisco Xavier, 1449-005 Lisbon, Portugal; 9 Medical Oncology Department, Unidade Local de Saứde São José, 1169-050 Lisbon, Portugal; 10 Centro Clínico Académico de Lisboa, 1169-056 Lisbon, Portugal; 11 Dev Group, Early Cancer Institute, Cambridge University, Cambridge CB2 0XZ, UK; 12 Faculty of Engineering, University of Porto, 4200-465 Porto, Portugal

**Keywords:** androgen deprivation therapy, prostate cancer, cognitive impairment, memory, executive function, scoping review

## Abstract

**Background:** Androgen deprivation therapy (ADT) is a primary treatment for prostate cancer (PCa) that effectively reduces androgen levels to suppress tumor progression. However, growing evidence suggests potential cognitive side effects, raising concerns about the long-term neurological consequences of this treatment. **Objective:** This scoping review aims to synthesize the existing evidence linking ADT to cognitive changes in men with PCa, identifying the key cognitive domains affected and outlining gaps in the existing literature. **Methods:** A systematic literature search was conducted according to the PRISMA-ScR guidelines in CINAHL, PubMed, Scopus, and Web of Science. Studies investigating cognitive function in ADT-treated PCa patients were included, covering randomized controlled trials (RCTs) and cohort, case–control, and cross-sectional studies. The extracted data included the study design, evaluated cognitive characteristics, measurement tools, and overall findings. **Results:** A total of 22 studies met the inclusion and exclusion criteria. Cognitive assessments varied across studies. While some studies reported cognitive impairments in ADT-treated patients—particularly in working, verbal, and visual memory and executive function—others found no significant effects. The variability in prostate cancer staging, epidemiological study designs, and treatment regimens; the exclusion of comorbid conditions; and the differences in assessment tools, sample sizes, and study durations hinder definitive conclusions about the cognitive effects of ADT. **Conclusions:** This scoping review highlights the heterogeneous and often contradictory evidence regarding ADT-associated cognitive dysfunction. While certain cognitive domains may be affected, methodological inconsistencies limit robust conclusions. Standardized cognitive assessments and longer longitudinal studies are required to clarify ADT’s role in cognitive decline. As the PCa survival rate increases with extended ADT use, integrating routine cognitive monitoring into clinical practice should be considered for PCa patients.

## Simple Summary

Hormone therapy is often used to treat prostate cancer, especially in men with advanced or high-risk disease. While this treatment can slow cancer growth, some patients report difficulties with memory, attention, or thinking after starting therapy. To better understand whether these changes are linked to hormone treatment, we reviewed all available research on how it may affect brain function. The results were mixed—some studies found signs of cognitive decline, while others did not. These differences may be due to the wide variety of study designs, patient groups, and testing methods. Our goals are to raise awareness that hormone therapy may influence cognitive function in some patients and to highlight the importance of regular brain health monitoring. We also aim to encourage future research that uses consistent methods to better understand and address these potential side effects. This review supports informed care and decision-making for men receiving hormone therapy.

## 1. Introduction

### 1.1. Prevalence and Treatment of Prostate Cancer

Prostate cancer (PCa) represents the second most prevalent cancer in men, with approximately 1.4 million new cases and 375,000 deaths reported in 2020 [1]. Over the past two decades, there has been a significant increase in the utilization of the prostate-specific antigen (PSA) test and subsequent biopsies, resulting in a notable rise in the number of PCa diagnoses [2]. At present, five-year survival rates approach 97%, creating a substantial population of long-term survivors who face extended treatment exposure and the associated long-term consequences [3].

Among the available treatments, androgen deprivation therapy (ADT) is a well-established systemic therapy that is particularly beneficial for advanced and metastatic PCa. While historically reserved for metastatic disease, ADT is increasingly used earlier in the disease course, especially in combination with radiotherapy for locally advanced cases [4]. A recent study showed that combining ADT with radiotherapy in locally advanced PCa prolonged survival and delayed the onset of symptoms by 18–24 months [5]. Consequently, the use of ADT has expanded, with an estimated 50% of PCa patients receiving it at some stage during their disease course [6].

The primary objective of ADT is to eliminate androgens—particularly testosterone—which are essential for the growth and proliferation of PCa cells [7]. ADT can be achieved either surgically, via bilateral orchiectomy, or pharmacologically, most commonly through the use of luteinizing hormone-releasing hormone (LH-RH) analogues, including certain agonists and antagonists (also known as gonadotropin-releasing hormone (GnRH) agonists/antagonists) [8]. These agents act through distinct mechanisms [9]: under physiological conditions, LH-RH is secreted in a pulsatile manner, stimulating the anterior pituitary to release luteinizing hormone (LH) and follicle-stimulating hormone (FSH) which, in turn, regulate testosterone production [10]. However, in pharmacological use, LH-RH agonists (e.g., leuprolide, goserelin, and triptorelin) are administered continuously, which initially stimulates the pituitary LH-RH receptors and causes a transient rise in LH, FSH, and testosterone levels—the so-called *androgen flare*. With continued exposure, this constant stimulation leads to receptor desensitization and downregulation, resulting in the suppression of LH and FSH secretion and a subsequent decrease in testosterone production. In contrast, LH-RH antagonists (e.g., degarelix and relugolix) directly block LH-RH receptors in the pituitary, thereby immediately suppressing LH and FSH secretion without triggering an initial flare. Both classes of agents ultimately lead to profound hypogonadism and medical castration [11].

### 1.2. Androgen Deprivation Therapy and Side Effects

The widespread use of ADT in PCa necessitates an understanding of its potential side effects. ADT profoundly impacts multiple organ systems beyond the area of tumor control. Key adverse effects include cardiovascular/metabolic disturbances, musculoskeletal deterioration, sexual dysfunction, and emerging cognitive concerns. A clear understanding of these complications is essential for optimizing survivorship care [12,13,14].

### 1.3. Cardiovascular and Metabolic Effects

Three large meta-analyses of observational studies have shown that prostate cancer patients treated with LH-RH agonists face significantly increased cardiovascular risks, including a 36–44% higher risk of cardiovascular death, a 19–57% greater risk of non-fatal cardiovascular disease, a 20–51% increased incidence of myocardial infarction, and up to a 51% increase in the risk of stroke, emphasizing the need for cardiovascular monitoring during ADT [15]. ADT also induces proatherogenic metabolic changes, even in men without prior risk factors. In one study, after ≥12 months of therapy, fasting insulin levels rose to 45 ± 7.25 µU/mL versus 24 ± 7.24 µU/mL in controls (*p* = 0.05), and 44% of patients developed diabetes-level fasting glucose. HOMA-IR scores increased from 6.0 ± 2.77 to 17.0 ± 2.78 (*p* < 0.01) independent of age and BMI, indicating that ADT-related hypogonadism itself contributes to this metabolic disruption and warrants routine glycemic surveillance [16].

### 1.4. Bone Density and Fracture Risk

ADT-mediated hypogonadism accelerates bone resorption via decreased estrogen conversion and increased osteoclast activity. Lumbar spine and femoral neck bone mineral density (BMD) has been shown to decline by 2–8% within the first year of therapy, with fracture hazards rising by 1.6-fold (adjusted HR 1.63; 95% CI 1.52–1.75) in real-world cohorts [17,18].

### 1.5. Muscle Mass and Body Composition

Men receiving ADT, especially for ≥ 6 months, exhibit significantly increased fat mass and reduced lean body mass compared to both PCa controls and healthy individuals. In a cross-sectional study, Clay et al. reported that long-term ADT was associated with notable shifts in body composition, independent of age or baseline adiposity [19]. Similarly, Basaria et al. found that men on ≥12 months of ADT showed marked increases in fat mass (*p* = 0.0001) and reductions in muscle strength and lean tissue, confirming that ADT-induced hypogonadism promotes adverse changes in body composition [20].

### 1.6. Sexual Dysfunction

Potosky et al. revealed that ADT led to a rise in the percentage of men who were sexually inactive, increasing from 45% to 80% after treatment with GnRH and from 48% to 83% following orchiectomy [21]. Beyond erectile dysfunction, patients experience decreased libido, anorgasmia, penile shortening, and testicular atrophy. Only ~20% of patients maintain any sexual function, underscoring the need for counseling and partner-inclusive support.

### 1.7. Cognitive Dysfunction

Interestingly, attention has recently been directed toward potential cognitive disturbances associated with ADT [22]. These cognitive disturbances may influence a patient’s capacity to make well-informed treatment choices or engage in occupational or intellectual activities, and they may also affect their overall quality of life. The expanding criteria for using ADT and its early application in PCa patients imply that, on average, patients will undergo ADT treatment for an extended period throughout the disease course. With the anticipated rise in the number of long-term cancer survivors, the lasting health effects of ADT, such as cognitive dysfunction, are expected to become more and more significant. A growing body of evidence suggests that androgens play an important role in cognitive functioning. Importantly, sex steroids influence cognition via two parallel receptor pathways: androgens bind androgen receptors (ARs) in neurons and glia to regulate synaptic plasticity, neurotrophic factor expression, and oxidative stress defenses; while a portion of testosterone is aromatized in situ to estradiol, activating estrogen receptors (ERα and ERβ) to modulate gene transcription and rapid signaling processes [23]. One key androgen metabolite, dihydrotestosterone (DHT), exerts neuroprotective effects by binding ARs, which are involved in synaptic plasticity, neuronal survival, and oxidative stress regulation—processes that are essential for cognitive health [24,25]. Recent research has further demonstrated that DHT administration attenuates neuroinflammation in microglia through inhibition of the p38 and p65 pathways, suggesting additional anti-inflammatory mechanisms beyond traditional receptor-mediated actions [26].

Westlye et al. demonstrated that ARs are abundantly expressed in the amygdala, brainstem, hypothalamus, and cerebral cortex—regions that are involved in both emotional regulation and cognitive processing [27]. The authors further observed the influence of anabolic androgen in diminishing the connection between the amygdala and the hippocampus, a link that is essential for maintaining cognitive function. Given that ADT significantly reduces circulating androgens, this disruption in amygdala–hippocampal connectivity may further exacerbate cognitive impairments in ADT-treated patients [28]. Consequently, the cognitive effects of ADT likely result not only from reduced estrogen production via aromatization, but also from the loss of direct AR-mediated signaling in critical brain regions.

Supporting this idea, epidemiological studies have linked low serum testosterone levels to poorer cognitive performance and an increased risk of age-related cognitive decline and dementia [29,30]. In a U.S. nationally representative sample from NHANES 2011–2014 (men aged ≥ 60 years), each higher quartile of free testosterone was associated with substantially lower odds of poor verbal memory (OR = 0.32; 95% CI: 0.17–0.61) and slowed processing speed (OR = 0.41; 95% CI: 0.17–0.96) [31]. Importantly, residual confounding by age-related comorbid conditions (e.g., obesity or metabolic syndrome) that both lower testosterone and impair cognition remains a concern [32,33]. Therefore, interventional studies investigating the effect of testosterone supplementation on cognition may provide more robust evidence. In this regard, randomized interventional trials provide more direct evidence. In a double-blind randomized controlled trial (RCT) including 57 healthy older men (mean age 67 ± 11 years), Cherrier et al. administered graded intramuscular testosterone (50, 100, or 300 mg) versus saline over 6 weeks. Subjects achieving a moderate rise in serum testosterone (~11–50 nmol/L above baseline) exhibited significant improvements in both verbal and spatial memory compared to those with minimal or supra-physiological elevations [34]. Studies involving patients with Alzheimer’s-related dementia or mild cognitive disorders have suggested that testosterone supplementation may improve certain aspects of cognitive function, particularly in hypogonadal men [35,36]. In a six-month RCT, Green et al. examined 82 men with advanced PCa and found that approximately half of those receiving androgen deprivation therapy (ADT)—including LH-RH analogues—exhibited clinically significant impairments in verbal memory, attention, or executive function. In contrast, none of the men under active surveillance (no treatment) showed cognitive decline over the same period [37]. While short-term studies like Green’s suggest selective cognitive deficits, longer-term data are more mixed. A 36-month prospective study by Alibhai et al. involving 241 men with non-metastatic PCa found no evidence of significant cognitive deterioration among ADT users compared to non-ADT PCa controls and healthy participants. Cognitive performance across eight domains remained stable over time, even after adjusting for ADT duration [38]. In contrast, Gunlusoy et al. conducted a prospective comparative study involving men with advanced PCa who were treated either with ADT or radical prostatectomy. Their findings indicated that ADT selectively impacted certain cognitive domains—specifically, language abilities, short-term memory, mental flexibility, and inhibitory control—while other domains remained unaffected [39]. These observations were further contextualized in a meta-analysis of 14 studies conducted by McGinty et al., who categorized cognitive outcomes into seven domains. The analysis revealed that men receiving ADT only performed significantly worse than controls or their own baselines in visuomotor tasks, with no consistent evidence of decline across other cognitive areas [40].

In summary, despite the growing body of research investigating the effects of ADT on cognitive function in patients with PCa, the findings to date remain inconclusive and, at times, conflicting. Some studies have identified specific domains of cognitive impairment associated with ADT, while others have found no significant changes. This inconsistency in the literature highlights the need for a more comprehensive and methodologically rigorous examination. Therefore, the objective of this scoping review is to synthesize and critically evaluate the existing evidence on the impact of ADT on cognition in patients with PCa. By integrating data obtained under various study designs and employing robust analytical techniques, we aim to provide a clearer understanding of the relationship between ADT and cognitive function, potentially guiding clinical practice and informing future research in this vital area of oncology care.

## 2. Methods

The guidelines on preferred reporting items for systematic reviews and meta-analyses extension for scoping reviews (PRISMA-ScR) were followed to report this systematic review and meta-analysis [41]. This scoping review was registered with INPLASY under the registration number INPLASY202570053.

### 2.1. Literature Search

A PCC framework comprising three distinct components of “Participant,” “Concept,” and “Context” was used to define the search phrase, as recommended in the Joanna Briggs Institute manual for scoping reviews [42] (Table 1).

We conducted a comprehensive search of the following databases from their inception to 2 February 2024: Scopus, Web of Science, PubMed, and the Cumulative Index to Nursing and Allied Health Literature (CINAHL). The search phrases are listed in the Supplementary Tables S1–S3.

### 2.2. Study Selection

**Figure 1:**
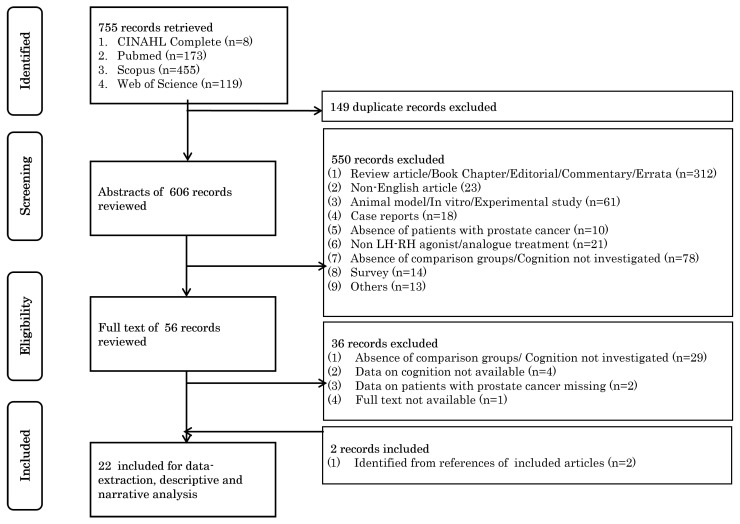
PRISMA flowchart for selection of studies.

Reference management software (EndNote 21.01.1, Build 17232) was used to manage and remove duplicate records. Two review authors (J.V.B. and G.F.) independently screened the titles, abstracts, or both of the remaining retrieved records to identify studies for further assessment. At this stage, irrelevant records such as review articles, book chapters, and editorials were excluded. Only articles published in English were considered. A further abstract review was conducted to exclude studies based on non-human populations and irrelevant study populations, interventions, or outcomes. Subsequently, the two review authors (J.V.B. and P.B.) independently reviewed the full texts of potentially relevant studies, mapped records to individual studies, and classified studies for inclusion if cognitive change was investigated and data were clearly extractable. Any discrepancies were resolved through consensus or, if needed, by consulting a third review author (D.G.). To identify other potentially eligible studies, we also searched the reference lists of the included studies. Reasons for excluding studies at each stage were documented, and the study selection process is presented in a PRISMA flow diagram (Figure 1).

### 2.3. Study Population and Setting

Studies were included if they enrolled adult men of any age with a histologically confirmed diagnosis of adenocarcinoma of the prostate who were receiving LH-RH analogues or agonists, including for metastatic or non-metastatic, localized, or advanced disease. Eligible studies had to assess cognitive performance in PCa and were conducted across all geographical settings and healthcare systems. Furthermore, studies had to be randomized trials, case-control studies, cohort studies (prospective or retrospective), or cross-sectional studies. The studies had to involve PCa.

### 2.4. Data Extraction

For studies that met the inclusion criteria, two review authors (J.V.B. and P.B.) independently extracted the following descriptive information: author and year, study design, country, PCa subtype, comparison groups, participant inclusion and exclusion criteria (e.g., age, comorbidities, and pretreatment status), interventions, sample size, study duration, or follow-up period (Table S1 in the Supplementary Materials). The authors further extracted the instruments used for cognitive performance assessment, key findings, and overall study conclusions, as shown in Table S2. Disagreements were resolved through discussion, consulting with a third review author (D.G.) if necessary.

## 3. Results

### 3.1. Study Selection

We retrieved a total of 755 records from our database searches. After removing the duplicates, 606 unique records remained. Following the screening of titles and abstracts, 56 potentially eligible records were identified, and their full texts were sought for detailed evaluation. Upon full-text review, 36 records were excluded as they did not meet the inclusion criteria. Additionally, we conducted a reference search of the remaining 20 records, which led to the identification of 2 additional relevant studies. In total, 22 studies were finally selected for data extraction to investigate the effects of LH-RH analogues on cognition [19,37,38,43,44,45,46,47,48,49,50,51,52,53,54,55,56,57,58,59,60,61]. The study selection process is summarized in the PRISMA flow diagram shown in Figure 1.

### 3.2. General Characteristics of the Studies

The extracted data were used to create a table detailing the general characteristics of the studies, as shown in Table 1.

### 3.3. Study Design

The comparison groups used to study the role of cognitive performance in the included studies were diverse. Seven studies compared PCa patients on ADT with those receiving non-ADT treatments in terms of cognition [19,38,43,46,50,55,59], while another six studies compared PCa patients on ADT with healthy controls (HCs) [47,48,49,56,58,60]. Three studies conducted multi-group comparisons across PCa patients on ADT, PCa patients receiving non-ADT treatments, and HC groups [19,37,45]. One study compared PCa patients on LH-RH analogues with another ADT option; namely, estradiol [44]. Additionally, five studies used a pre–post design to assess changes in cognition over time in PCa patients undergoing ADT [51,52,53,54,61]. Two studies compared patients on short-term ADT to those on long-term ADT [57]. Concerning the study design, we identified 3 randomized controlled trials (RCTs) [37,44,45], 15 cohort studies (14 prospective and 1 retrospective) [38,43,46,47,49,50,51,52,53,54,55,58,59,60,61], 1 case–control study [48], and 3 cross-sectional studies [19,56,57]. The majority of studies were conducted in populations of European descent, with eight studies from Europe [44,47,51,52,54,55,56,57] and nine from North America (including six from the USA and three from Canada) [19,38,43,46,48,49,58,59,61], highlighting a significant predominance of Western populations. Only two studies were conducted in populations of Asian ancestry (both in Japanese populations) [53,60], and three were conducted in Australasian populations (including two from Australia and one from New Zealand) [37,45,50].

### 3.4. Inclusion and Exclusion Criteria

The inclusion and exclusion criteria varied substantially across the included studies, highlighting their heterogeneous designs and highly specific objectives. Only six studies explicitly specified age criteria. One study included participants aged 18 years or older [52], two included those aged 50 years or older [19,56], two set the age criterion at 55 years or older (one focusing on ages 55–85) [57,61], and one included participants aged 75 years or older [51,52]. Of the 22 studies, 13 excluded participants with conditions likely to confound cognitive outcomes, such as dementia, psychiatric or neurodegenerative disorders, cerebral diseases, or brain cancer [37,38,43,45,48,49,52,54,56,57,58,59,60]. Several studies also employed highly specific exclusion criteria—such as hemoglobin levels of <10 mg/dL [61], severe lower urinary tract symptoms (LUTS, associated with significant disease burden or comorbidities) [37,45], or the presence of another active malignancy [38,43]. While one study excluded participants who had undergone prostatectomy to eliminate the confounding effects of prior surgery [59], another excluded hypogonadal HC participants [19], ensuring that the control group maintained a distinct hormonal profile. Some studies also required participants to have basic education or native language proficiency to improve their comprehension of the cognitive assessment tools and enhance the reliability of self-reported data [38,43,48,49,56]. Treatment history was another critical factor, with many studies excluding participants who had received prior hormonal therapy [37,45], were undergoing chemotherapy [54,58], or had been castrated at baseline [52], thereby minimizing confounding factors that could independently affect cognition. A few studies restricted inclusion to patients who had not received LH-RH analogues within a specified period before starting ADT [52,57], ensuring a consistent hormonal baseline, while others focused on patients receiving ADT for at least 3 months as adjuvant therapy [49] or for biochemical relapse following prostatectomy or radiotherapy (to ensure stable hormonal conditions and a meaningful assessment) [46,49,58]. Concerning the stage of disease progression, 12 studies investigated only those patients with non-metastatic PCa [19,38,43,46,47,48,49,53,55,58,59,61], including a study that focused exclusively on localized cases [47]. Another nine studies included a mixture of non-metastatic and metastatic cases [37,44,45,50,51,52,54,56,60], with two focusing solely on non-localized cases [37,45].

### 3.5. Conceptual and Operational Definitions of Cognitive Performance

The conceptual and operational definitions of cognitive performance (CP) in the included studies are presented in Table S1. Several cognitive domains are particularly important, with specific assessment tools frequently being employed to evaluate the cognitive effects of ADT in PCa. However, there was no consistency across studies, as each study employed its own set of cognitive domains and assessment tools.

### 3.6. General Cognitive Function

General cognitive function was most commonly assessed using the mini-mental state examination (MMSE) tool. This tool appeared in five studies and was widely used to screen overall cognitive impairment, due to its simplicity and reliability; however, it has limitations in terms of detecting subtle cognitive changes [54,55,57,60,61]. Another tool, the cognitive functioning subscale of QLQ-C30 (EORTC), was applied in three studies to evaluate quality-of-life-related cognitive function [44,50,51].

### 3.7. Memory (Verbal and Visual)

Memory was a key domain assessed in most ADT studies, with a variety of tools being used to evaluate the different aspects of memory. The California verbal learning test (CVLT) and its short-form version (CVLT-SF) were among the most frequently applied tools, appearing in three studies [38,43,61]. These tests evaluate verbal learning, recall, and recognition, focusing on how individuals encode, retain, and retrieve verbal information over multiple trials. The Wechsler memory scale (WMS) was utilized in two studies to provide a broader assessment of memory impairments, measuring both visual and verbal memory through tasks involving immediate and delayed recall, recognition, and working memory [37,47]. The digit span test (forward and backward) was employed in two studies to assess working memory and attention span, with the backward version adding a layer of complexity by testing both cognitive flexibility and mental manipulation [38,43]. Tests such as the Rey–Osterrieth complex figure test and the brief visual memory test (BVMT) were also employed to assess visual recall and visual–spatial relationships, with the former emphasizing organizational strategies and spatial memory and the latter focusing on shape recall [37,48]. Additionally, another study applied ad hoc visual memory tests to assess spatial and visual memory impairments, often exploring specific visual–spatial relationships or navigation abilities that standardized tests might not address [52].

### 3.8. Attention and Processing Speed

Attention and processing speed were commonly evaluated across studies, with the trail-making test (TMT)—particularly its Part B—being used in three studies. This test assesses cognitive flexibility, processing speed, and task-switching abilities, as participants alternate between numbers and letters in sequence. It is especially effective for identifying deficits in attention and multitasking [37,38,43]. Another study used the digit symbol substitution test (DSST) to measure sustained attention, psychomotor speed, and processing efficiency, requiring participants to match symbols with numbers under time constraints. This test is sensitive to cognitive slowing and provides a measure of overall processing speed [19].

### 3.9. Executive Function

Executive function, an important cognitive domain, includes skills such as problem-solving, cognitive flexibility, and inhibitory control. Several neuropsychological tests were employed to evaluate this domain. The Stroop test, which was used in two studies, measures cognitive flexibility, interference control, and selective attention by requiring participants to name the ink color of incongruent color words. This test is particularly effective at detecting deficits in managing conflicting information [45,58]. The N-Back task and stop-signal task were used in one study to measure real-time cognitive control and error correction [59]. The N-Back task assesses working memory by requiring participants to identify matches between the current stimulus and one presented *n* number of steps earlier. The stop-signal task evaluates inhibitory control by instructing participants to perform a primary task while occasionally requiring them to withhold their response to a “stop” signal. The trail-making test Part B and the D-KEFS color word inference test were used to examine higher-order executive functions, including problem-solving and cognitive flexibility [38,43].

### 3.10. Visuospatial Abilities

Visuospatial abilities were evaluated using a range of tools that measure spatial reasoning, perception, and construction. The judgment of line orientation test, which appeared in three studies, assesses spatial orientation and perception by requiring participants to identify the angles of intersecting lines. This test is particularly effective for detecting impairments in understanding spatial relationships [38,43,52]. The mental rotation task, used in three studies, evaluates spatial reasoning by testing the participant’s ability to mentally rotate two- or three-dimensional objects and identify whether they matched a reference figure [47,52,58]. Another study employed the block design test, which measures spatial construction abilities by having participants arrange blocks to replicate geometric patterns, testing their visuospatial integration and problem-solving [58].

### 3.11. Contents and the Effect of ADT Intervention

#### 3.11.1. ADT Intervention

The majority of studies (13 out of 22) specifically analyzed the use of LH-RH analogues as the primary ADT intervention [37,44,45,47,48,49,51,52,53,54,55,60,61], underscoring their predominant role in the management of PCa across the included research (Table 1). Among these, three studies specifically examined GOS [47,55,59] and two investigated LEU [53,61]. However, the individual contributions of specific LH-RH analogues were not explicitly detailed in the remaining studies, possibly due to the limited sample sizes in each subgroup. The remaining nine studies may have included patients treated with other forms of ADT, such as AA or orchiectomy. Among the studies reporting LH-RH analogue subtypes, leuproline (LEU) was the most commonly administered treatment, followed by goserelin (GOS) and triptorelin (TRP). We observed a wide variation in study durations, ranging from a minimum follow-up of just 3 months for individuals with PCa to an average follow-up of 6.4 years in one study [47,51].

#### 3.11.2. Effect of ADT Intervention

The overall findings on the effects of ADT on CP in PCa patients, as reported in the included studies, are summarized in Table S1. To ensure methodological rigor and enhance interpretability, the results are grouped by principal cognitive domains: memory (working, verbal, and visual), attention and processing speed, executive function, and global cognitive performance. These domains reflect the key neuropsychological constructs that were most frequently assessed across studies. Where applicable, we also highlight between-group comparisons (e.g., ADT vs. non-ADT or healthy controls) and within-subject analyses (pre- vs. post-ADT exposure) using validated cognitive assessment instruments and standardized follow-up intervals.

### 3.12. Memory Domains (Working, Verbal, and Visual Memory)

Several studies have reported ADT-related impairments in memory. In a Canadian one-year prospective study, Alibhai et al. compared 77 ADT-treated non-metastatic PCa patients (94.8% on LH-RH analogues) with 82 non-ADT patients and 82 HCs [43]. ADT-treated patients showed significant declines in working memory, as measured by the Digit Span Backward Test (DSBT; *p* = 0.029) and the Spatial Span Backward Test (SSBT; *p* = 0.031). The same group also reported improvements in visuospatial ability in the non-ADT group, specifically via the card rotation test (CRT; *p* = 0.034), as well as sustained global cognitive improvement over a three-year follow-up period (*p* = 0.003), particularly in terms of visuospatial functioning (*p* = 0.031) [38]. The decline in working memory has also been corroborated by Ihrig et al., who reported deficits in working memory performance in a German cohort undergoing ADT [56]. Green et al., in an Australian RCT involving 48 ADT-treated and 14 non-ADT patients, used the auditory verbal learning test (AVLT) and observed significantly worse performance in the ADT group after 12 months (*p* = 0.014) [45]. Visual memory findings were less consistent. Morote et al., in a six-month Spanish study with 308 patients, reported no significant changes in visual memory or other domains [52]. Conversely, Sánchez-Martínez et al., using the MMSE and B-Cog tools with 33 metastatic and non-metastatic patients, observed significant post-ADT declines in verbal and non-verbal memory (*p* = 0.035 and *p* = 0.001, respectively) [54].

These collective findings suggest that ADT may negatively influence working and verbal memory, although findings regarding visual memory remain less consistent.

### 3.13. Attention and Processing Speed

Attention-related deficits have been variably reported. Clay et al. evaluated visuomotor performance using the digit symbol substitution test (DSST) in 42 non-ADT patients, 12 long-term ADT patients (mean 30.7 months), and 25 short-term ADT patients (mean 3.7 months). No significant group differences were found [19]. However, Ihrig et al. reported significant deficits in word fluency and processing speed when using the trail-making test (TMT) and auditory verbal learning test (AVLT) in 139 ADT-treated PCa patients (mean ADT duration: 3.5 years for LH-RH analogues and 0.4 years for abiraterone) [56]. In a prospective UK study by Jenkins et al., 47 localized PCa patients treated with GOS were compared to 38 HCs over three months. In this study, 47% of the ADT patients exhibited declines in at least one test (e.g., intelligence, memory, or processing speed), compared to only 18% of controls (*p* = 0.033) [47].

These results suggest that although attentional and processing speed impairments are not universal, certain patient subsets—particularly those with longer ADT duration—may be at elevated risk.

### 3.14. Executive Function

Executive functioning was explicitly evaluated in fewer studies. Sánchez-Martínez et al. observed significant executive function decline (*p* = 0.001) when using the B-Cog in a Spanish cohort [54]. Other studies employed self-reported tools such as the EORTC QLQ-C30 cognitive functioning subscale, which includes only two items assessing concentration and memory. For instance, Lebret et al. assessed 500 French patients (mean age 80.4 years) using the EORTC QLQ-C30 after 4.1 months of treatment and reported no cognitive decline [51]. Similarly, Okamoto et al. conducted a study in 45 Japanese patients with localized or locally advanced PCa, and they found no significant change in MMSE scores after 6 and 12 months of ADT [53].

The discrepancy between objective and self-reported measures, along with differences in the sensitivity of the instruments used, limits the drawing of definitive conclusions. Nonetheless, the presence of executive impairments in objective assessments such as the B-Cog suggests that a subset of patients may experience domain-specific executive dysfunction under ADT.

### 3.15. Global Cognitive Performance

Global cognition has been assessed in both retrospective and prospective studies. Alibhai et al. observed cognitive improvement in non-ADT patients over a three-year period (*p* = 0.003), particularly in visuospatial functioning (*p* = 0.031) [38]. Tan et al. followed 50 non-metastatic patients in the US over 12 months and observed improvements in verbal learning and memory using the CVLT-SF, without declines in MMSE scores [61]. Joly et al. evaluated 57 non-metastatic ADT patients and 51 HCs using the MMSE, HCSS, and FACT-Cog instruments over 1.8 years and found no significant group differences [49]. A retrospective study in New Zealand (75 ADT vs. 131 non-ADT) and another in the UK (315 LH-RH vs. 412 estradiol) also showed no differences in EORTC QLQ-C30 scores after six months [44,50].

These diverse findings point to substantial heterogeneity in the global cognitive effects of ADT, likely influenced by cohort characteristics, treatment duration, and cognitive assessment methods.

## 4. Discussion

The present scoping review aimed to identify and describe the existing literature investigating the effects of LH-RH analogues on cognitive function in men with PCa. A total of 22 studies were identified, covering a broad range of epidemiological study designs, including RCTs and PCSs. In addition, we observed considerable heterogeneity in patient characteristics, the treatments administered, sample sizes, study durations, and assessment tools. Furthermore, the findings of this scoping review underscore a complex and often contradictory relationship between ADT and cognitive impairment in PCa.

While some studies reported a decline in cognitive functioning in patients treated with ADT, others found no significant differences between ADT-treated patients and controls. Nevertheless, several consistent themes emerged. First, potential cognitive deficits were noted in specific domains, such as working memory, verbal memory, or visuomotor tasks, among those patients receiving ADT compared to those not receiving ADT or HCs. Second, PCSs that examined longitudinal changes in cognition exclusively within LH-RH analogue-treated cohorts without a control group generally did not detect such effects [51,52,53,54,61]. Third, although most of the included ADT-based studies used LH-RH analogues, we found relatively few investigations focusing on these analogues exclusively, making it challenging to ascertain whether any observed deficits were attributable specifically to LH-RH analogues in such studies. Collectively, these three patterns support the idea that, while evidence for ADT-related cognitive impairment exists, substantial heterogeneity in the study designs complicates efforts to draw definitive conclusions as to the effects of LH-RH analogues on cognitive performance.

During this scoping review, it became clear that the absence of standardized definitions for cognitive assessments led researchers to measure cognitive functioning in diverse ways. Some incorporated comprehensive test batteries targeting memory, processing speed, and executive control, while others relied exclusively on brief screening tools such as the MMSE [53,55], and some even used the cognitive functioning subscale of the QLQ-C30 [44,51]. While the MMSE may be less sensitive to mild or domain-specific effects, test batteries are better equipped to detect such changes [62]. Hence, relying solely on screening instruments like the MMSE may lead to under-reporting of the prevalence and severity of cognitive impairments. Similarly, the cognitive functioning subscale of the QLQ-C30 provides valuable subjective insights into how patients perceive their cognitive abilities, but should not be considered a standalone indicator of actual cognitive performance [63]. Our scoping review underscores the importance of incorporating comprehensive multi-domain test batteries in future studies.

The variability in study populations—specifically, the inclusion of men with localized PCa versus mixed cohorts of non-metastatic and metastatic patients—introduces critical challenges in interpreting the relationship between ADT and cognitive dysfunction. Patients with localized disease are often younger, healthier, and receive ADT as an adjuvant or neoadjuvant therapy (e.g., combined with radiation). Their ADT duration is typically shorter (6–36 months), and they may have fewer comorbidities (e.g., cardiovascular disease or diabetes) that may independently affect cognition. For example, Alibhai et al. (2017) reported minimal cognitive changes in non-metastatic patients after 36 months of ADT [38]. In contrast, metastatic PCa patients are older, more likely to receive lifelong ADT, and often have advanced disease or comorbidities that exacerbate cognitive decline. Metastasis itself may contribute to systemic inflammation, indirectly impairing cognition [64]. For instance, Ihrig et al. (2023) reported significant working memory deficits in metastatic patients [56]. Furthermore, such studies may have been more sensitive in detecting these effects, as evidenced by the reported significant impact of ADT on cognition. Metastatic cohorts are also frequently treated with ADT combined with novel hormonal agents (e.g., abiraterone or enzalutamide) or chemotherapy, which may themselves induce cognitive changes [65]. Such confounding factors may not have been fully separated from the effects of ADT. In summary, the majority of studies combined localized and metastatic cohorts, which could have obscured the true cognitive effect of ADT.

Different epidemiological study designs—such as RCTs, PCSs, and cross-sectional studies—introduce variations in the strength of evidence, generalizability, and susceptibility to bias. RCTs provide the highest level of evidence in determining causal relationships due to their ability to minimize confounding variables. In this review, three RCTs were identified that assessed the cognitive effects of ADT [37,44,45]. These trials implemented randomization, ensuring balanced groups at baseline and reducing the selection bias. However, limitations such as small sample sizes and relatively short follow-up durations (ranging from 6 to 12 months) may have restricted their ability to detect the long-term cognitive effects of ADT. Despite this restriction, Green et al. (2002) reported significant impairments in verbal memory and executive function in ADT-treated patients after six months, reinforcing the concern that hormonal deprivation affects cognitive performance [37]. In contrast, while PCS designs allow for the evaluation of temporal changes and long-term outcomes, they remain vulnerable to high drop-out rates, confounding factors, and a lack of generalizability. For example, Alibhai et al. (2017) failed to detect significant declines in working memory over 36 months of ADT exposure [38]. However, they acknowledged that their findings might not be representative of the general older male population, as the participants were in relatively good health aside from their PCa and, on average, had high levels of formal education. It is possible that ADT use in more cognitively vulnerable individuals (e.g., those with prior strokes or early dementia) or in less well-educated men may result in greater cognitive decline. Lastly, although cross-sectional studies (CSs) such as that by Ihrig et al. (2023) may suggest potential cognitive differences, their inability to establish causality limit their interpretability [56].

The selection of exclusion criteria in studies investigating cognitive changes associated with ADT involves a delicate trade-off between internal validity (isolating the specific effects of ADT) and external validity (ensuring that the findings apply to real-world populations). Striking this balance is particularly challenging in neurocognitive research, where comorbidities are prevalent yet may independently influence cognitive performance. Studies such as that conducted by Chao et al. (2012) excluded patients with cardiovascular disease or neurological conditions, isolating the effects of ADT but creating “idealized” samples [59]. For instance, excluding hypertensive patients (a condition common in older adults) may lead to underestimation of the interaction between ADT and cerebrovascular dysfunction [59]. Jim et al. (2010) included patients with neuropsychiatric conditions and found that 42% of ADT patients had multi-domain impairment vs. 19% of HCs, suggesting that neuropsychiatric conditions may compound ADT risks [48]. However, studies of neuropsychiatric conditions, such as that conducted by Morote et al. in 2017, reported fewer deficits without any significant association of ADT use with cognitive decline [52].

In keeping with the methodology of scoping reviews, this synthesis did not undertake a formal critical appraisal of each included study; thus, no definitive grading of evidence quality was performed. Consequently, readers should interpret any trends in cognitive outcomes with caution, bearing in mind that studies of weaker design, minimal sample size, or inconsistent measures might over- or underestimate the true impact of ADT. Additionally, limiting our selection to English-language papers raises the possibility that pertinent data from non-English publications were overlooked. Finally, while the database search was deliberately broad, the inclusion of additional gray literature or the expansion of search parameters in other languages could potentially help to elucidate further relevant evidence.

Despite this being a scoping review demonstrating variations in research quality, the collective findings underscore several actionable points for epidemiologists, clinicians, and investigators. As PCa survival rates rise, there is a need for ongoing surveillance of cognitive health in men on ADT. From a clinical perspective, heightened vigilance for potential cognitive side effects is warranted, especially given the long duration of ADT in many PCa treatment regimens. Addressing these effects demands a multidisciplinary approach wherein urologists, oncologists, psychologists, and allied health professionals jointly monitor patients’ cognitive trajectories. This could mean implementing standardized cognitive screens with validated, domain-specific instruments to catch early indications of cognitive strain. As most research on PCa-related cognitive deficits remains in its early stages, large-scale, prospective, or longitudinal investigations that systematically document changes in cognitive performance over multiple timepoints—from pre-ADT baseline through months or years of therapy—are crucial for dissecting the direct impacts of ADT on other confounding variables influencing cognition in older adults. There is a pressing need to harmonize outcome measures into a core set of cognitive domains that would enable meaningful cross-study comparisons and meta-analyses. Epidemiologists, clinicians, and researchers must collaborate and coordinate their efforts to achieve meaningful progress. Such efforts will help to optimize treatment decisions, align therapeutic strategies with patient preferences, and expand our understanding of how ADT can be administered without unduly compromising cognitive well-being.

## 5. Conclusions

In summary, while this scoping review did not establish a causal relationship between ADT and cognitive decline, the accumulated evidence suggests that certain cognitive domains—particularly working memory, verbal learning, and executive functioning—may be vulnerable in patients receiving long-term ADT. The variability in study designs, patient populations, and assessment tools complicates definitive interpretation. Nevertheless, the emerging patterns support the need for increased clinical awareness, the implementation of routine cognitive monitoring, and investment in high-quality prospective studies. Standardization of cognitive outcome measures and stratification by ADT type and PCa stage will be crucial to advance research in the field and guide patient-centered decision-making.

## Figures and Tables

**Table 1:** PCC framework used to define the search phrase and the selection of studies.

Component	Details
Population (P)	Men with a confirmed histological diagnosis of adenocarcinoma of the prostate, including metastatic or non-metastatic, localized, or advanced prostate cancer.
Concept (C)	Cognitive change.
Context (C)	Treatment with LH-RH analogues or agonists.

## Supplementary Materials

The following supporting information can be downloaded at: https://www.mdpi.com/article/10.3390/cancers17152501/s1, Table S1: General characteristics of the included studies (n = 22); Table S2: Cognitive performance-related characteristics and results in the included studies (n = 22).
