# Supplementary material for: Cognitive Impairment in Prostate Cancer Patients Receiving Androgen Deprivation Therapy: A Scoping Review
Source: Cancers (Basel). 2025 Jul 29;17(15):2501. doi: 10.3390/cancers17152501 (PMC12346850; doi:10.3390/cancers17152501)
Supplement: Supplementary file 1 [file cancers-17-02501-s001.zip › Supplementary Table S1.pdf]

Supplementary Table S1.

Author (year)	Comparison (Cases vs Controls)	Study design	Country	PCa type	Inclusion/Exclusion criteria	Treatment administered	Sample size	Study/ Follow-up duration (months)
**ADT vs. Non-ADT**								
Wiechno et al. (2015) [55]	PCa on LH-RH analogue/agonist vs PCa without ADT	PCS	Poland	Localized or locally advanced	Cases: Men receiving adjuvant hormonotherapy with LH-RH analogue/agonist monotherapy after radical radiotherapy completion. (Inc)<br>Controls: Men not receiving adjuvant monotherapy, without an LH-RH analogue/agonist after radical radiotherapy completion (Inc)<br>Men with evidence of progression after radical radiotherapy completion (Exc)	GOS* (Monotherapy) (Cases), Non-ADT (Controls)	GOS: 88<br>Non-ADT: 61	Not mentioned
Alibhai et al. (2010) [43]	PCa on ADT vs PCa without ADT vs HC	PCS	Canada	Non-metastatic	Presence of another active malignancy (Exc)<br>Life expectancy < 1 year (Exc)<br>Presence of a major neuropyschiatruc abnormality (Exc)	LH-RH analogue/agonist or AA or Non-ADTor HC (Cases/Controls)	ADT: 77 (LH-RH analogue/antagonists: 73, AA: 4)<br>Non-ADT: 82<br>HC: 82	12

					Suspicion of cognitive impairment (MMSE <24 of 30) (Exc)<br><8 years of education (Exc)			
Alibhai et al. (2017) [38]	PCa on ADT vs PCa without ADT vs HC	PCS	Canada	Non-metastatic	Presence of another active malignancy (Exc)<br>Life expectancy < 1 year (Exc)<br>Presence of a major neuropyschiatruc abnormality (Exc)<br>Suspicion of cognitive impairment (MMSE <24 of 30) (Exc)<br><8 years of education (Exc)	LH-RH analogue/agonist or AA or Non-ADT or HC (Cases/Controls)	ADT: 77 (LH-RH analogue/antagonists : 73, AA: 4)<br>Non-ADT: 82<br>HC: 82	Median: 36 (ADT treatment: 29.3)
Chao et al. (2012) [59]	PCa on ADT vs PCa without ADT	PCS	USA	Non-metastatic	Cases: Men prescribed ADT—either as adjuvant treatment or because of biochemical recurrence (Inc)	GOS* (Cases), Non-ADT (Controls)	ADT: 15	6

Controls: Patients with non-metastatic PCa who had never been treated with ADT with matching based on age and level of education (Inc)	Non-ADT: 15
Eligible for fMRI (Inc)<br>Participants who had a prostatectomy were at least 3 months from their surgery and had fully recovered before study entry (Inc)<br>Active second malignancy (Exc)<br>Eastern Cooperative Oncology Group Performance Status >1 (Exc)<br>any significant cardiovascular conditions (e.g., unstable angina, pacemaker);<br>hepatic, renal, or neurological disease;<br>any investigational agents (Exc)<br>A score of less than 27 out of 30 on the mini-mental state examination (MMSE) (Exc)	

					History of axis-I psychiatric or substance (excluding nicotine) use disorders (Exc)			
Clay et al. (2007) [19]	PCa on short-term ADT vs Pca on long-term ADT vs PCa without ADT vs HC<br><br>PCa without ADT vs PCa on short-term ADT<br>PCa without ADT vs PCa on long-term ADT<br>PCa on short-term ADT vs PCa on long-term ADT<br>PCa on short-term ADT vs HC<br>PCa on long-term ADT vs HC	CS	USA	Non-metastatic	Community-dwelling ≥50 years of age (Inc)<br>Any disease or on medication known to affect bone and mineral metabolism (Exc)<br><br>HC: Hypogonadal (Exc)	LH-RH analogue/agonist or AA or Orch (ADT) or Non-ADT or No treatment (Cases/Controls)	Short-term ADT: 25<br><br>Long-term ADT: 12<br><br>Non-ADT: 42<br><br>HC: 20	Long-term ADT: Mean: 30.7, SD: 28.9 (Duration of treatment)<br><br>Short-term ADT: Mean: 3.7, SD: 1.3 (Duration of treatment)
Karunasinghe et al. (2016) [50]	PCa on ADT vs PCa without ADT	RCS	Newzealan d	Metastatic and non-metastatic	No specific criteria	LH-RH analogue/agonist or AA or Orch or Estrogen (Cases);	ADT: 75 (Approx****)	Not specified

						Non-ADT (Controls)	Non-ADT: 131	
Green et al. (2002) [37]	PCa on LH-RH analogue/agonist vs PCa on AA vs PCa without ADT (CCM) (Baseline vs Follow-up) (No specific distinction between cases and controls)	RCT	Australia	Extraprostatic	Previous hormonal therapy (Exc)<br>Psychiatric impairment (Exc)<br>Severe LUTS (IPSS>7) (Exc)<br>Abnormal serum testosterone (Exc)	LEU* or GOS* or CPA** or Non-ADT (Cases/Controls)	All: 65<br>LEU: 19<br>GOS: 20<br>CPA: 11<br>Non-ADT: 15	6
Green et al. (2004) [45]	PCa on LH-RH analogue/agonist vs PCa on AA vs PCa without ADT vs HC (Community based)	RCT	Australia	Non-localized	Previous hormonal therapy (Exc)<br>Psychiatric impairment (Exc)<br>Severe LUTS (IPSS>7) (Exc)	LEU* or GOS* or CPA** or Non-ADT (CCM) or No treatment (Cases/Controls)	LEU: 18<br>GOS: 19<br>CPA: 11	12

					Abnormal serum testosterone levels (Exc)		Non-ADT: 14<br>HC:15	
Herr et al. (2000) [46]	PCa on ADT vs PCa without ADT vs PCa without treatment	PCS	USA	Non-metastatic	Asymptomatic locally advanced, nonmetastatic PCa or PSA relapse after curative local therapy	LH-RH analogue/agonist or AA or Orch (ADT) or Non-ADT or No treatment (Cases/Controls)	ADT: 79<br>Non-ADT: 36<br>No treatment: 29	12
Gilbert et al. (2017) [44]	PCa on LH-RH analogue/agonist vs PCa on tE2	RCT	UK	Locally advanced or metastatic	Locally advanced or metastatic PCa, and a treatment plan for indefinite ADT in the metastatic setting or ≥3 years for locally advanced disease.	LH-RH analogue/agonist (Cases); tE2 (Controls)	LH-RH analogue/agonist: 315<br>tE2: 412	6
**ADT vs. HC**								
Jim et al. (2010) [48]	PCa on LH-RH analogue/agonist vs HC	matched CC	USA	Non-metastatic	**PCa on LH-RH analogue/agonist:** Were able to speak and read English (Inc)	LEU* or GOS* (Cases); No treatment (Controls)	LEU: 30<br>GOS: 18	Not applicable

Had at least an eighth grade education (Inc)<br>Receiving treatment with either an LH-RH analogue/agonist agonist alone or combined AA/LH-RH analogue/agonist agonist therapy (Inc)<br>Had been treated continuously with either an LH-RH analogue/agonist agonist alone or combined AA/LH-RH analogue/agonist agonist therapy for at least 6 months prior to assessment (Inc)<br>Non-demented (Inc)<br>**HC:**<br>Were able to speak and read English (Inc)<br>Had at least an eighth grade education (Inc)<br>No history of cancer diagnosis other than basal cell skin carcinoma (Inc)<br>Within five years of age of the patient participant to whom they were being matched (Inc)	HC: 48

					Had the same educational level as the patient participant to whom they were being matched (i.e., ≤12 years versus >12 years) (Inc)<br>Non-demented (Inc)			
Jenkins et al. (2005) [47]	PCa on LH-RH analogue/agonist vs. HC	PCS	UK	Localized	No specific criteria	GOS* (Cases); No treatment (Controls)	GOS: 32<br>HC: 18	3 to 5
Ihrig et al. (2023) [56]	PCa on ADT vs HC (from reference population)	CS	Germany	Metastatic	>50 years (Inc)<br>Cases: PCa received one or more ADT courses (Inc)<br>Poor german language skills (Exc)<br>Visual or hearing impairment (Exc)<br>Known brain metastases (Exc)	LH-RH analogues/agonists or BIC** or ENZ** or AA (Cases), No information on controls	LH-RH analogues/agonists: 54<br>BIC: 37<br>ENZ: 21<br>AA: 27<br>HC: Not available	LH-RH analogues/agonists: 42.4 (Duration of treatment)<br>BIC: 8 (Duration of treatment)<br>ENZ: 5 (Duration of treatment)<br>AA: 10 (Duration of treatment)

					Prior diagnosis of severe neurological or psychiatric disease (Exc)			
Joly et al. (2006) [49]	PCa on LH-RH analogue/agonist vs. HC	PCS	Canada	Localized non-metastatic	PCa with ADT for at least 3 months as adjuvant therapy, or for biochemical relapse (increasing PSA) after prostatectomy or radiotherapy (Inc)<br>Individuals with no major illness, no pre-existing psychiatric history and not on neuroleptic drug (Inc)<br>Individuals with fluent english (Inc)<br>Individuals with ability to perfrom physical tests (Inc)	LH-RH analogue/agonist or AA (Cases), No treatment (Controls)	LH-RH analogue/agonist: 48<br><br>LH-RH analogue/agonist and AA: 9<br><br>HC: 51	Median: 21.6, Range: 4.8 to 88.8
Cherrier et al. (2003) [58]	PCa on ADT vs HC	PCS	USA	Non-metastatic	PCa with increasing PSA (biochemical relapse) following primary therapy (radiation, brachytherapy or prostatectomy) for which androgen suppression therapy is indicated<br>Pain related to PCa (Exc)	LEU* and FLU** or BIC** (Cases), No treatment (Controls)	ADT: 19<br><br>HC: 15	12 (9 months of treatment and after 3 months off treatment)

					History of psychiatric illness, dementia, central nervous system metastasis (Exc)<br>History of systemic chemotherapy and current renal or hepatic dysfunction (Exc)			
Yamamoto et al. 2023 [60]	PCa on LH-RH analogue/agonist vs HC	PCS	Japan	Localized or metastatic	MMSE<24 (Exc)	LH-RH analogue/agonist (Cases), No treatment (Controls)	ADT: 43<br>HC: 34	Median: 36, Range: 18-36
**ADT pre vs post**								
Sanchez-Martinez et al. (2021) [54]	PCa on LH-RH analogue/agonist (Baseline vs Follow-up)	PCS	Spain	Metastatic or non-metastatic	Receiving any other chemotherapy treatment (Exc)<br>Known cognitive deterioration (Exc)<br>Change in health status after baseline assessment which could influence sleep quality, mood, or cognitive performance (Exc)	LEU* or TPR* (Cases/Controls)	All: 33<br>LEU: 9<br>TPR: 24	12
Morote et al. (2017) [52]	PCa on LH-RH analogue/agonist (Baseline vs Follow-up)	PCS	Spain	Localized, Locally advanced, metastatic	≥ 18 years with histologically-confirmed PCa (Inc)	LH-RH analogue/agonist (Cases/Controls)	All: 308	6

					On LH-RH analogue/agonist analogue for ≥ 6 months (Inc)<br>Prior LH-RH analogue/agonist analogue during the preceding 6 months (Exc)<br>Presence of neuro-psychiatric pathology (Exc)<br>Castrated as baseline (Exc)			
Tan et al. (2013) [61]	PCa on LH-RH analogue/agonist (Baseline vs Follow-up)	PCS	USA	Non-metastatic	Age ≥55 years (Inc)<br>Hemoglobin levels<10 mg/dL (Exc)	LEU* (Cases/Controls)	All: 50	12
Okamoto et al. (2015) [53]	PCa on LH-RH analogue/agonist (Baseline vs Follow-up)	PCS	Japan	Localized and locally adavanced	6-month neoadjuvant ADT with radiation therapy, followed by LEU monotherapy (Inc)	LEU* (Monotherapy) (Cases/Controls)	All: 45<br>`	12
Lebret et al. (2014) [51]	PCa on LH-RH analogue/agonist (Follow-up vs Baseline)	PCS	France	Metastatic and non-metastatic	PCa ≥ 75 years (Inc)<br>PCa initiated on LH-RH agonist/analogue, not treated wtih LH-	LH-RH analogue/agonist or combination of LH-RH analogue with AA (Cases/Controls)	<784	Mean: 4.1, SD: 2.3, Range: 3 to 6 (Approx****)

					RH agonist/analogue for last 12 months (Inc)			
Shah et al. 2018 [57]	Early ADT (Treatment ≤ 4 months) vs. Late ADT (Treatment > 4 months)	CS	UK	No mention	Age between 55 and 85 years (Inc) History of neuropsychiatric pathology, stroke, traumatic brain injury or neurodegenerative disorders and associated dementia or delirium (Exc) Cognitive impairment prior to starting ADT with LH-RH analogue/agonist or history of receiving ADT with LH-RH analogue/agonist prior to current treatment (Exc)	LH-RH analogue/agonist (Cases/Controls)	Early ADT: 8<br>Late ADT: 12	Median: 36, Range: 18-36

*LH-RH/GnRH analogue or agonist

**AA: Antiandrogen

***LH-RH/GnRH antagonist

****Based on all the participants in the study (including those not considered for the present review)

Abbreviations (Arranged alphabetically)

AA: Abiraterone acetate (androgen biosynthesis inhibitor)

ADT: Androgen depriviation therapy

BIC: Bicalutamide
CC: Case-control
CCM: Close clinical monitoring
CDR: Clinical dementia rating
CI: Confidence interval
CP: Cognitive peformance
CPA: Cyproterone acetate
CS: Cross-sectional
ENZ: Enzalutamide
Exc: Excluded
FLU: Flutamide
GOS: Goserelin
HC: Healthy control
HIS: Histlerin
Inc: Included
IPSS: International prostate symptom score
KET: Ketoconazole
LEU: Leuprolide
LH-RH/GnRH: Luteinizing hormone-releasing hormone/Gonadotropin-releasing hormone
LUTS: Lower urinary tract symptoms
MMSE: Mini-Mental State Examination
NIL: Nilutamide
Orch: Orhiectomy
PCa: Men with Prostate cancer
PCS: Prospective cohort study
RCS: Retrospective cohrot study
RCT: Randomized controlled trial
RR: Relative risk
SD: Standard deviation
TPR: Triptorelin
