# Supplementary material for: Cognitive Impairment in Prostate Cancer Patients Receiving Androgen Deprivation Therapy: A Scoping Review
Source: Cancers (Basel). 2025 Jul 29;17(15):2501. doi: 10.3390/cancers17152501 (PMC12346850; doi:10.3390/cancers17152501)
Supplement: Supplementary file 1 [file cancers-17-02501-s001.zip › Supplementary Table S2.pdf]

**Supplementary Table S2.**

| Author (year) | Outcome assessment | Findings | Conclusion |
|---|---|---|---|
| **ADT vs. Non-ADT** | | | |
| Wiechno et al. (2015) [55] | MMSE | PCa on LH-RH analogue/agonist: CP score: 27.59, Non-ADT: CP score: 27.41 (P=0.551) | The study concluded that LH-RH analogues do not have a negative impact on cognitive function. |
| Alibhai et al. (2010) [43] | NAART: General intelligence<br>Digit Span Forward and Spatial Span Forward: Immediate span of attention<br>Trail Making Test part A: Processing speed<br>COWT and Animal Fluency: Verbal fluency<br>Card Rotations and Judgment of Line Orientation: Visuospatial ability<br>CVLT: Verbal learning and memory<br>Brief Visual Memory Test: Visual learning and memory<br>Digit Span Backward, Digit Span Forward, Spatial Working Memory Task Errors, Conditional Associative Learning Test: Executive functions, working memory<br>Trail Making Test part B, D-KEFS<br>Color Word Inference Test: Executive functions, cognitive flexibility | At 6 months, PCa on ADT showed no change in any of the cognitive scores compared to PCa on non-ADT and HC (P>0.05)<br>At 12 months, PCa on ADT showed significant decline in digit span forwared, spatial span backward and lesser gain in card rotations compared to PCa on non-ADT and HC (P=0.029, 0.031 and 0.034 respectively).<br>No significant change in ≥ 1/2 cognitive tests | The study failed to find consistent evidence suggesting that 12 months of ADT use negatively impacts cognitive function in PCa. |
| Alibhai et al. (2017) [38] | Digit Span Forward and Spatial Span Forward: Immediate span of attention<br>Trail Making Test part A: Processing speed | PCa on non-ADT showed no improvement in cognitive scores compared to Pca on ADT and HC in 13 out of 14 cognitive scores (P>0.05)<br>PCa on non-ADT showed significant improvement in card rotations compared to PCa on ADT and HC (P=0.031). | The use of ADT for up to 36 months does not appear to be effect cognitive decline. |

| | | | |
|---|---|---|---|
| | COWT and Animal Fluency: Verbal fluency<br>Card Rotations and Judgment of Line Orientation: Visuospatial ability<br>CVLT: Verbal learning and memory<br>Brief Visual Memory Test: Visual learning and memory<br>Digit Span Backward, Digit Span Forward, Spatial Working Memory Task Errors, Conditional Associative Learning Test: Executive functions, working memory<br>Trail Making Test part B, D-KEFS Color Word Inference Test: Executive functions, cognitive flexibility | PCa on non-ADT showed significant improvement in global cognitive z-score ompared compared to PCa on ADT users and HC (P=0.020)<br>No significant decline in ≥ 1/2 cognitive tests in PCa on ADT compared to PCa on non-ADT and HC | |
| Chao et al. (2012) [59] | N-back task (0-,1-,2-back): Working memory<br>Stop-signal task (Go success rate, Stop success rate, stop signal reactime time, post-error slowing): Cognitive control | No significant difference in CP between PCa on ADT compared to PCa without ADT (P>0.05) | The evidence does not support an association between treatment with ADT for six months and cognitive decline. |
| Clay et al. (2007) [19] | DSST (WAIS-R): Visuomotor performance (Response speed, sustained attention, visual spatial skills, and set shifting) | Overall ANOVA P=0.0148<br>Difference: -5.1, 95% CI: -12.9 to 2.8 (P>0.05)<br>Difference: -3.1, 95% CI: -9.3 to 3.1 (P>0.05)<br>Difference: -2.9, 95% CI: -9.6 to 3.8 (P>0.05)<br>Difference: 2.2, 95% CI: -5.7 to 10.1 (P>0.05)<br>Difference: 0.2, 95% CI: -6.3 to 6.7 ( (P>0.05) | CP varied among patients on long-term ADT, short-term ADT, those not receiving ADT, and healthy controls but was not significantly lower in any specific group, suggesting that ADT had little effect on CP. |
| Karunasinghe et al. (2016) [50] | Cognitive functioning subscale of QLQ-C30 (EORTC) | Long-term ADT (>6 months) vs non ADT: Relative difference: -36.5, 95% CI: 78.5 to 5.6 (P=0.0736)<br>Short-term ADT (<6 months) vs non ADT: Relative difference: 13.4, 95% CI: -29.0 to 55.7 (P=0.430) | ADT treatment showed no decline in cognitive function over both short-term (<6 months) and long-term (>6 months) durations. |
| Green et al. (2002) [37] | WMS-R: Visual and verbal memory Indices | GOS: WMS-R verbal memory (Difference between 6 months and baseline score): 2.6 to 9.5 (P<0.05) | Six months of ADT monotherapy may lead |

| | | | |
|---|---|---|---|
| | AVLT: The sum of words recalled from five-list learning trials<br>Rey-Osterrieth Complex Figure Test: 30-min delayed recall in | GOS: WMS-R Visual memory (Difference between 6 months and baseline score): 1.1 to 5.9 (P<0.05)<br>GOS: AVLT (Difference between six months and baseline score): -5.9 to -0.5 (P<0.05)<br>PCa without ADT :WMS-R verbal memory (Difference between 6 months-baseline score): 4.3 to 16.2 (P<0.05)<br>48% of PCa on ADT showed reliable decline in at least one cognitive task (P<0.01)<br>Group-Time interaction: P=0.008 | to impairments in memory, attention, and executive functioning. |
| Green et al. (2004) [45] | Visual, verbal memory Index, AVLT (trials 1-5): Memory<br>Attention and concentration index, digit symbol (coding task), trail making test part A, trail making test part B, COWA: Attention<br>Stroop test neutral words, stroop test colour words, Rey figure copy: Executive functions | **AVLT: Group-Time interaction P<0.01**<br>At baseline, 6 and 12 months: HC showed significantly better performance compared to PCa (P=0.024, P=0.001, and P=0.009 repectively)<br>At 12 months, PCa on LEU showed significantly better performance compared to PCa on GOS (P=0.008)<br>At 12 months, PCa without ADT showed significantly better performance than PCa on ADT (P=0.014)<br>**Digit symbol: Group-Time interaction P<0.05**<br>At baseline, 6 and 12 months: HC showed significantly better performance compared to PCa (P<0.05)<br>**Stroop test color words: Group-Time interaction P=0.087**<br>At 6 and 12 months: HC showed significantly better performance compared to PCa (P=0.046 and P=0.003, respectively) | The study observed adverse effects on cognitive function in PCa treated with 12-months of LEU and GOS |
| Herr et al. (2000) [46] | Intrusion subscale of the IES | ADT vs. Local treatment vs No treatment: 1.8 vs 1.7 vs 1.6 (P=0.20) | This study found no clinical evidence of a relationship between LHRH agonists/analogues and cognitive decline. |

| | | | |
|---|---|---|---|
| | | ADT vs. (Local treatment + No treatment): 1.8 vs 1.6 (P=0.16)<br>LEU+FLU vs LEU vs Orch: 2.3 vs 1.8 vs 1.2 (P=0.01) | |
| Gilbert et al. (2017) [44] | Cognitive functioning subscale of QLQ-C30 (EORTC): | Mean difference in 6-month score: 1.9, 95% CI: -1.8 to 5.5 (P=0.32) | The evidence does not support an association between LH-RH agonists/analogues and cognitive decline. |
| **ADT vs. HC** | | | |
| Jim et al. (2010) [48] | HVLT-R: Verbal memory<br>COWA: Verbal fluency<br>BVMT-R: Visuospatial memory<br>Card rotation test: Visuospatial abilities<br>SDMT: Executive functions | All treatments: No significant differences in CP score means (P>0.05)<br>All treatments:: 42% of PCa showed greater overall impairments vs 19% of HC showed greater overall impairment (P<0.05)<br>(Overall impairment: % of individuals with impaired performace in two or more tests) | The evidence from the present study indicates that LH-RH agonist therapy for PCa is linked to notable impairments in cognitive function. |
| Jenkins et al. (2005) [47] | National Adult Reading Test: Intelligence<br>Phonemic verbal fluency task: Verbal ability | 47% of PCa and 17% of HC (OR: 4.412, P=0.033) showed cognitive reliable decline at 3 months in at least one task<br>34% of PCa and 28% of HC showed reliable cognitive decline on completing GOS treatment in at least one task (OR: 1.37, P=0.631). | The findings indicate that short-term LH-RH therapy for early-stage PCa has a limited and |

| | | | |
|---|---|---|---|
| | Rey AVLT: Verbal memory<br>Complex Figure Task: Visual memory<br>Computerized mental rotation task: Visual spatial memory<br>Wechsler Memory Scale III tasks: the digitspan task and the spatial-span task: Working memory<br>KDCT: Processing speed | Group-Time interaction: P>0.05 | modest impact on cognitive function in men. |
| Ihrig et al. (2023) [56] | WIWO: Lexical word fluency<br>AWLT: Long term verbal memory and verbal learning ability<br>TMT: Attention, processing speed, mental ability<br>NBV working memory task: Verbal working memory | All cognitive domains except learning total showed significant reduction in scores in PCa on ADT compared to HC (WIWO vs HC: P<0.001, AWLT (Short-term delayed retrieval) vs HC: P<0.01, TMT-A vs HC, P<0.001, TMT-B vs HC, P<0.05, NBV (correct) vs HC: P<0.001, NBV (incorrect) vs HC: P<0.01) | ADT for metastatic PCa was associated with neurocognitive deficits across multiple domains, with language abilities and processing speed being the most commonly affected. Despite this, no uniform pattern of cognitive impairment was observed. |
| Joly et al. (2006) [49] | Folstein MMSE: Cognitive decline<br>HSCS: Verbal memory, language, visual-motor, spatial, attention and concentration, and self-regulation and planning<br>FACT-Cog: Self reported measure of cognitive function: Mental acuity, attention and concentration, memory, verbal, fluency, functional interference, deficits observed by others, change from previous functioning and impact on QL | No significant difference in HSCS (and its domains) and FACT-Cog between PCa on ADT and HC (P>0.05) | This study found no impact of ADT on physical or cognitive function in patients with prostate cancer. |
| Cherrier et al. (2003) [58] | Route test: Spatial memory<br>Block design and mental rotation: Spatial ability | Significant impovement in verbal-recall in the PCa on ADT compared to those on HC (P<0.05)<br>Significant impovement in spatial memory in HC compared to those on ADT (P<0.05) | These findings indicate that 9 months of combined androgen blockade improved |

| | | | |
|---|---|---|---|
| | Proactive interference and story recall: Verbal memory<br>Verbal fluency: Language<br>Stroop test and self-ordered pointing test: Executive function | | verbal memory but had a negative impact on spatial ability in patients with prostate cancer. |
| Yamamoto et al. 2023 [60] | MMSE | No significant difference in MMSE between PCa on ADT and HC at any of the follow-ups (6, 12 and 36 months) (P>0.05) | Over a 36-month follow-up period, ADT was not associated with cognitive decline. |
| **ADT pre vs post** | | | |
| Sanchez-Martinez et al. (2021) [54] | MMSE and its dimensions (orientation, spatial orientation, immediate recall, attention and calculation, delayed recall, language)<br>BCog and its dimensions (communication, attention, recent memory, concentration, remote memory, orientation, calculation and executive function). | All treatments: Calculation dimension of Bcog: Mean: 4.2, SD: 2.3 at baseline; Mean: 5.2, SD: 2.2 at 12 months (P=0.001) | This study found no clinical evidence of a relationship between ADT using LHRH agonists/analogues and cognitive decline over a 12-month follow-up period. |
| Morote et al. (2017) [52] | WAIS III digit span subtest: Working memory<br>Ad hoc visual memory test: Visual memory<br>Judgement of line orientation test and mental rotation of three-dimensional objects test: Visospatial ability<br>WAIS III matrix reasoning test: nonverbal analytical reasoning | All treatments: No significant change for majortity of PCa (245/308), 18.2% showed signifiant decline and 18.8% showed significant improvement in one of the CP test. | CP in patients with PCa does not seem to be negatively impacted by 6 months of LHRH analogue therapy. |
| Tan et al. (2013) [61] | MMSE: Cognitive decline<br>CVLT-SF: Memory and learning (Dimensions: Learning, Delayed free | No significant change in MMSE scores at 2,4 and 12 months compared to baseline (P>0.05 across all the comparisons).<br>Significant improvement in CVLT-SF scores at 2,4 and 12 months compared to baseline (P<0.01 across all the compaisons). | LEU therapy was not linked to any decline in cognitive or memory function. |

| | | | |
|---|---|---|---|
| | recall, Cued recall, Rcognition discriminability) | | |
| Okamoto et al. (2015) [53] | MMSE | No significant change in MMSE scores at 6 and 12 months compared to baseline (P>0.05 across all the comparisons). | LH-RH agonist monotherapy did not lead to a decline in cognitive functions. |
| Lebret et al. (2014) [51] | Cognitive functioning subscale of QLQ-C30 (EORTC) | Mean difference: 0.9, 95% CI: -0.4 to 2.2 (P=0.248) | This study indicates that cognitive function remains unaffected during 3–6 months of LH-RH agonist therapy in older PCa. |
| Shah et al. 2018 [57] | MMSE | No significant difference (P=0.610) | No association was found between cognitive function scores on the MMSE and the duration of LH-RH agonists/analogues in PCa. The MMSE may not be an effective tool for assessing cognitive function in this population. |

# When possible, only data on LH-RH analogue/agonist was extracted from publications, Any ADT treatments may be in combination with other ADT or non-ADT treatment, unless stated as monotherapy

*LH-RH/GnRH analogue or agonist

**AA: Antiandrogen

***LH-RH/GnRH antagonist

****Based on all the participants in the study (including those not considered for the present review)

**Abbreviations (Arranged alphabetically)**

AA: Abiraterone acetate (androgen biosynthesis inhibitor)

AD: Alzheimer's disease
ADT: Androgen depriviation therapy
AVLT: Auditory-Verbal Learning Test
AWLT: Auditory Word List Learning Test
BCog: The Brief Scale for Cognitive Evaluation
BIC: Bicalutamide
BT: Brachytherapy
BUS: Buserelin
BVMT-R: Brief Visuospatial Memory Test-Revised
CC: Case-control
CCM: Close clinical monitoring
CDR: Clinical dementia rating
CI: Confidence interval
COWA: Controlled Oral Word Association test
CP: Cognitive peformance
CPA: Cyproterone acetate
CS: Cross-sectional
CVLT-SF: California Verbal Learning Test- Short Form
DEG: Degarelix
D-KEFS: Delis-Kaplan Executive Function System
DSST: Digit Symbol Substitution Test
ENZ: Enzalutamide
Exc: Excluded
FACT-Cog: Functional Assessment of Cancer Therapy-Cognitive Function
FLU: Flutamide
GOS: Goserelin
HC: Healthy control
HIS: Histlerin

HR: Hazard ratio
HRQol: Health related quality of life
HSCS: High sensitivity cognitive screen
HVLT-R: Hopkins Verbal Learning Test-Revised
IES: Impact of Event Scale
Inc: Included

IPTW: inverse probability of treatment weighting
IQR: Interquartile range
KCDT: Kendrick Assessment of Cognitive Ageing.
KET: Ketoconazole
LEU: Leuprolide
LH-RH/GnRH: Luteinizing hormone-releasing hormone/Gonadotropin-releasing hormone
Local therapy: RT or RP or BT
LUTS: Lower urinary tract symptoms
MMSE: Mini-Mental State Examination
NAART: The North American Adult Reading Test
NBV: N-Back verbal
NIL: Nilutamide
OR: Odds ratio
Orch: Orhiectomy
PCa: Men with Prostate cancer
PCS: Prospective cohort study
Q1: Quartile 1
Q3: Quartile 3
QL: Quality of life
QLQ-C30 (EORTC): The European Organisation for Research and Treatment of Cancer (EORTC) Quality of Life Questionnaire-Core 30 (QLQ-C30)
RCS: Retrospective cohrot study
RCT: Randomized controlled trial

RP: Radical prostatectomy
RR: Relative risk
RT: Radiation therapy
SD: Standard deviation
SDMT: Symbol Digit Modalities Test
TBI: Traumatic brain injury
tE2: transdermal estradiol
TMT: Trail Making Test
TPR: Triptorelin
TURP: Transurethral resection of prostate
WAIS III: Wechsler Adult Intelligence Scale III
WAIS-R: Wechsler Adult Intelligence Scale-Revised
WIWO: Vienna Word Fluency Test
WTS: Vienna Test System
